# Integrating landfill bioreactors, partial nitritation and anammox process for methane recovery and nitrogen removal from leachate

**DOI:** 10.1038/srep27744

**Published:** 2016-06-09

**Authors:** Faqian Sun, Xiaomei Su, Tingting Kang, Songwei Wu, Mengdong Yuan, Jing Zhu, Xiayun Zhang, Fang Xu, Weixiang Wu

**Affiliations:** 1 Institute of Environmental Science and Technology, Zhejiang University, Hangzhou 310058, China; 2 Quzhou Environmental Sanitation Department, Quzhou 324000, China; 3 Zhejiang Institute of Microbiology, Hangzhou 310012, China

## Abstract

A new process consisting of a landfill bioreactor, partial-nitritation (PN) and the anammox process has been developed for landfill leachate treatment. In this study, the landfill bioreactor exhibited excellent performance in methane-rich biogas recovery, with a specific biogas yield of 0.47 L gas g^−1^ COD and methane percentages of 53–76%. PN was achieved in the aerobic reactor by high free ammonia (101 ± 83 mg NH_3_ L^−1^) inhibition for nitrite-oxidizing bacteria, and the desired PN effluent composition (effluent nitrite: ammonium ratio of 1.1 ± 0.3) was controlled by adjusting the alkalinity concentration per unit of ammonium oxidized to approximately 14.3 mg CaCO_3_ mg^−1^ N in the influent. The startup of anammox process was successfully achieved with a membrane bioreactor in 160 d, and a maximum nitrogen removal rate of 216 mg N L^−1^ d^−1^ was attained for real landfill leachate treatment. The quantitative polymerase chain reaction results confirmed that the cell-specific anammox activity was approximately 68–95 fmol N cell^−1^ d^−1^, which finally led to the stable operation of the system.

Landfilling of municipal solid waste is still one of the most attractive options for waste management^1^. One of the greatest environmental concerns in landfill sites is the production of municipal landfill leachate. The production of such leachate presents high variability, and the leachate is typically characterized by high concentrations of ammonium and COD, which has a low biodegradable content^2^. Consequently, the low carbon-to-nitrogen (C/N) ratio of the leachate makes it expensive to treat with the conventional nitrification-denitrification process owing to its high oxygen demand and the addition of an external carbon source.

A treatment based on partial nitritation (PN) and an autotrophic anaerobic ammonium oxidation (anammox) process would be an attractive alternative to treat landfill leachate owing to the reduced aeration demand and lower organic carbon requirements^3^. During PN, approximately half of the ammonium in the wastewater is oxidized to nitrite by ammonia-oxidizing bacteria (AOB) under aerobic conditions; then, anammox bacteria (AMX) convert the remaining ammonium and the produced nitrite directly to nitrogen gas^4^. Previous studies have demonstrated that the presence of biodegradable organic matter could negatively inhibit the anammox process. To apply the anammox process to the treatment of high strength landfill leachate, biodegradable COD should be removed to avoid its inhibitory effect on the subsequent anammox process. Several studies report the use of landfills as bioreactors to be one of the most attractive and practical methods for treating landfill leachate, with the potential to accelerate waste biodegradation and recover energy^5^,^6^. Therefore, a new process, consisting of a landfill bioreactor for methane recovery and PN/anammox for nitrogen removal, would be a promising and cost-effective method for treating municipal landfill leachate.

To facilitate highly efficient nitrogen removal through the PN/anammox process, ammonium in the wastewater should be first partially oxidized to nitrite to produce a suitable mixture with 1.32 moles NO_2_^−^ per mole of NH_4_^+^ in the PN process^7^. Therefore, both the nitrification pathway and ammonium conversion efficiency must be controlled simultaneously. Generally, PN could be obtained by enabling a higher growth rate of AOB relative to that of nitrite-oxidizing bacteria (NOB). The main driving forces are the inhibition of NOB by free ammonia (FA)^8^, the low concentration of dissolved oxygen (DO)^9^, the short sludge retention time (SRT)^10^ or a combination of these factors. However, these driving forces also have their drawbacks and make it difficult to achieve a stable and efficient PN process. It has been shown that the use of bicarbonate control technique is feasible to achieve PN performance^11^. Still, under the conditions of varying influent composition, there are few successful reports about its application to landfill leachate treatment in the long-term. In addition, the combined effects of bicarbonate limitation and inhibition factors on the achievement of PN in landfill leachate treatment have not yet been fully investigated.

In the anammox process, one of the main challenges is the long start-up time because of the slow growth rate of AMX. Achieving the appropriate biomass retention by minimizing biomass washout in reactors becomes a crucial factor for reactor startup and stable operation. To date, various bioreactor configurations have been applied to implement anammox, such as moving bed biofilm reactor (MBBR)^12^, upflow anaerobic sludge blanket (UASB) reactor^13^, sequencing batch reactor (SBR)^14^ and rotating biological contactor^15^,^16^. However, a fraction of biomass is inevitably washed out with the effluent in all these systems, especially for unstable periods due in many cases to overloads, which provoke the biomass flotation^17^. Fortunately, membrane bioreactor (MBR), which is effective in the complete retention of suspended biomass, could be developed as an ideal reactor to initiate and maintain the anammox process^18^. Wyffels, *et al*.^19^ firstly used anammox MBR to treat sludge reject water and showed that nitrogen removal efficiency was 82% at the nitrogen loading rate between 650 and 1100 mg N L^−1^ d^−1^. Trigo, *et al*.^17^ successfully achieved the startup of anammox process in a MBR with a maximum nitrogen removal rate (NRR) of 710 mg N L^−1^ d^−1^ after approximately one year. However, few studies have been conducted on the operation of the anammox process in MBRs for landfill leachate treatment. Previous studies have mainly focused on nitrogen removal performance of anammox in the treatment of mature landfill leachate with low C/N ratio^20^,^21^,^22^. Wang, *et al*.^1^ reported that in a single partially aerated full-scale bioreactor for the treatment of mature landfill leachate, combined PN and anammox contributed to 68% of the total nitrogen (TN) removal. Li, *et al*.^21^ reported that during long-term operation, the anammox reactor treating mature landfill leachate can only stably operate under nitrogen load of 1000 mg N L^−1^ d^−1^ with 85% of nitrogen removal. Up to date, systematic investigations of the operating conditions of integrated landfill bioreactors, PN and anammox MBR for landfill leachate treatment and evaluations of the performance of the combined process are still scarce.

The primary aim of this paper is (1) to investigate the feasibility of achieving carbon and nitrogen removal from the integrated landfill bioreactor, PN and anammox process; (2) to investigate the methane recovery capacity of a landfill bioreactor; (3) to achieve the long-term production of a suitable mixture of ammonium and nitrite through the PN process; and (4) to assess performance during startup and maintenance of the anammox process in landfill leachate treatment and explore the abundance of functional microbial populations involved.

## Results and Discussion

### Methane recovery from a landfill bioreactor

**Figure 1:**
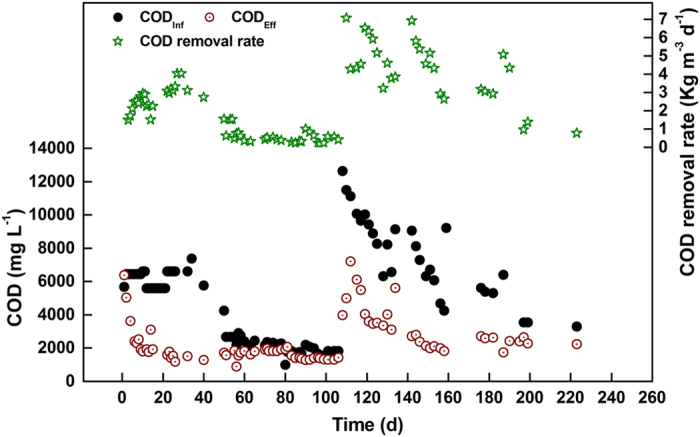
COD removal performance in the landfill bioreactor.

Figure 1 shows the system performance related to COD removal during operation. On days 1 to 70, the influent flow rate was approximately 6.5 L d^−1^. From day 71 onward, the influent flow rate was adjusted to 12 L d^−1^. Figure 1 illustrates that when the COD concentrations in the feed varied from 1532 to 12633 mg L^−1^, the effluent COD concentration ranged from approximately 883 to 7200 mg L^−1^. With the increasing COD concentration and increasing flow rate of the influent, the COD removal rate gradually increased to its highest level of 6.91 kg m^−3^ d^−1^ on day 142. The correlations between COD removal and biogas production were also investigated, and the biogas production rate calculated from the slope of the curve indicated that the specific biogas yield was 0.47 L gas g^−1^ COD (see Supplementary Fig. S1). The values are higher than those reported for anaerobic SBR treatment of landfill leachate (0.4 L gas g^−1^ COD) or anaerobic digestion treatment of mixed sludge (0.4 L gas g^−1^ COD)^23^, suggesting that organic matter may be degraded in the landfill refuse. The methane percentage in the biogas ranged from 53% to 76% indicating a specific methane production rate of 0.25–0.35 L CH_4_ g^−1^ COD. Similar results have been reported by other researchers^24^,^25^. Therefore, a landfill bioreactor can efficiently recover methane-rich biogas for *in-situ* organic removal.

### Achievement of long-term PN

**Figure 2:**
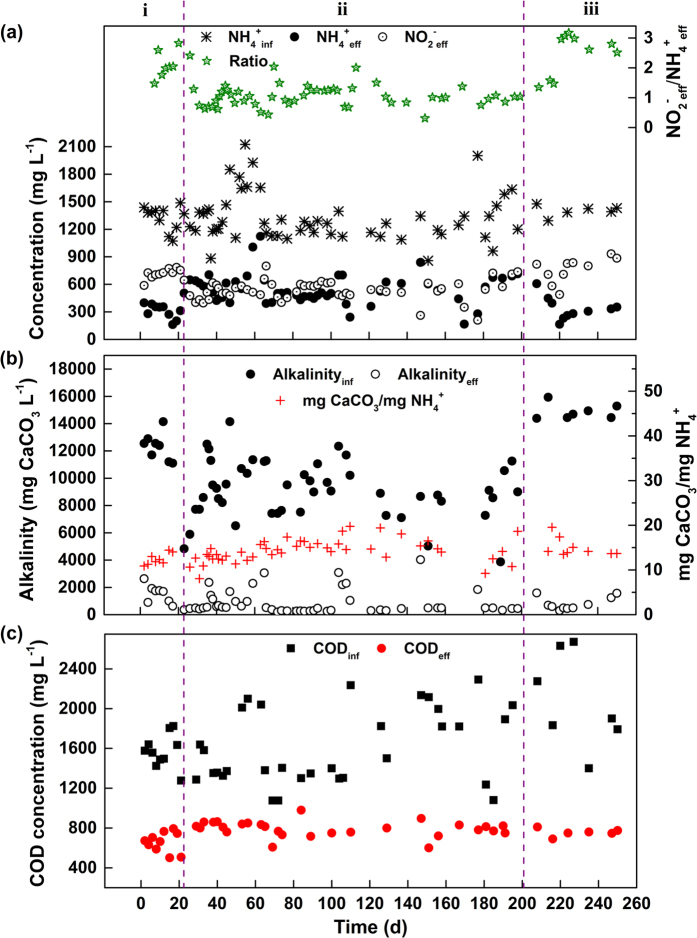
Performance of partial nitritation in the anoxic-aerobic reactors: (**a**) influent ammonium, effluent ammonium and nitrite together with the nitrite-to-ammonium molar ratio; (**b**) influent and effluent alkalinity concentration as well as the ratio of decreased alkalinity to oxidized ammonium; and (**c**) influent and effluent COD concentrations.

The reactor was successfully operated for 250 days treating municipal landfill leachate. Figure 2a presents the evolution of influent ammonium, effluent ammonium and nitrite together with the nitrite-to-ammonium ratio in the aerobic reactor. Figure 2b shows the influent and effluent alkalinity evolution as well as the ratio between decreased alkalinity and oxidized ammonium. The entire operation period was divided into three phases according to different feeding alkalinity conditions. During the first 23 days (Phase i), the reactor was fed with anaerobically treated leachate without adjusting the alkalinity. As shown in Fig. 2a, a high proportion of ammonium oxidized to nitrite was reached, and no significant nitrate production was detected. The nitrite-to-ammonium ratio produced in the aerobic reactor varied between 1.5 and 4.5, with an average of 2.4. In this period, as presented in Fig. 2b, the available bicarbonate in the influent was sufficient for ammonium oxidation, and the effluent alkalinity concentration was approximately 1500 mg L^−1^. To reduce the bicarbonate used for ammonium oxidation, sulfuric acid was added to the influent during Phase ii (days 24–200) to achieve the desired effluent composition. When the influent ammonium concentration was 1327 ± 262 mg N L^−1^, the effluent ammonium and nitrite concentrations were 526 ± 122 and 549 ± 108 mg N L^−1^, respectively. This method resulted in the production of a suitable influent (nitrite: ammonium ratio = 1.1 ± 0.3) to feed the subsequent anammox reactor. With the exception of a few abnormal days (days 36, 63–65, 104–108 and 147), the influent alkalinity was sufficient for ammonium oxidation and the effluent alkalinity was higher than 2000 mg L^−1^. During the majority of this period, the influent alkalinity was limited, with above 90% of alkalinity removed from the system, and the effluent alkalinity was lower than 500 mg mg CaCO_3_ L^−1^. The consumption of alkalinity per unit of ammonium oxidized was calculated to be 14.3 ± 2.5 mg CaCO_3_ mg^−1^ N, which was lower than the theoretical stoichiometric amount required to achieve the desired effluent composition. In Phase iii (days 201–250), the influent was again recovered without alkalinity adjustment, and the influent alkalinity concentration was always higher than 14000 mg CaCO_3_ L^−1^. The nitrite-to-ammonium ratio increased considerably to 2.6 ± 0.6, with the average ammonium and nitrite concentrations reaching approximately 308 and 752 mg N L^−1^.

During the entire period, the consumption of alkalinity fit well with the unit of ammonium oxidation, with an average value of 14.0 mg CaCO_3_ mg^−1^ N, demonstrating that bicarbonate is the key to controlling the conversion ratio of ammonium to nitrite. The results were consistent with Shanahan and Semmens^26^ who reported that the bicarbonate alkalinity of the influent strongly influenced the nitrification performance and a molar stoichiometric ratio of HCO_3_^−^/NH_4_^+^-N = 2.0 was required to neutralize the acidity produced during nitrification. The obtained value was also close to that reported by Ganigue, *et al*.^27^. These researchers found that the ratio of alkalinity consumed to ammonium oxidized was equal to 14.3 mg CaCO_3_ mg^−1^ N. Using this method, the percentage of ammonium oxidized to nitrite in the reactor could be controlled by the alkalinity concentration in the influent. In this study, PN via the nitrite pathway was the most effective part of the nitrification process. The specific oxygen uptake rates also showed that ammonium oxidation was the dominant process (see Supplementary Fig. S2). The reason for this could be that when high bicarbonate concentration was used as the alkalinity source, AOB were dominant but NOB were eliminated in the reactor^11^. Another reason could be that FA could create a possible inhibitory environment for NOB in the first unit of the aerobic reactor. The concentration of FA in the first unit of the aerobic reactor was calculated to be 101 ± 83 mg NH_3_ L^−1^, which was considerably higher than any inhibitory value for NOB (1–5 mg NH_3_ L^−1^) reported by Abeling and Seyfried^28^. Therefore, high FA concentrations drove stable PN to inhibit NOB more than AOB. In the PN process, the favorable conditions of both FA inhibition and alkalinity limitation guaranteed the stable effluent back to the anammox reactor.

With regard to organic matter, appropriately 200 mg L^−1^ COD was removed in anoxic reactor and the majority of the biodegradable organic matter was removed in the aerobic reactor (Fig. 2c). Under the steady-state conditions, the effluent COD concentration ranged from 608 to 863 mg L^−1^ with an average value of 786 mg L^−1^. As a fraction of landfill leachate in the influent was from old landfill cells, the COD present in the effluent of the aerobic reactor was mainly refractory^29^.

### Nitrogen removal from the anammox reactor

**Figure 3:**
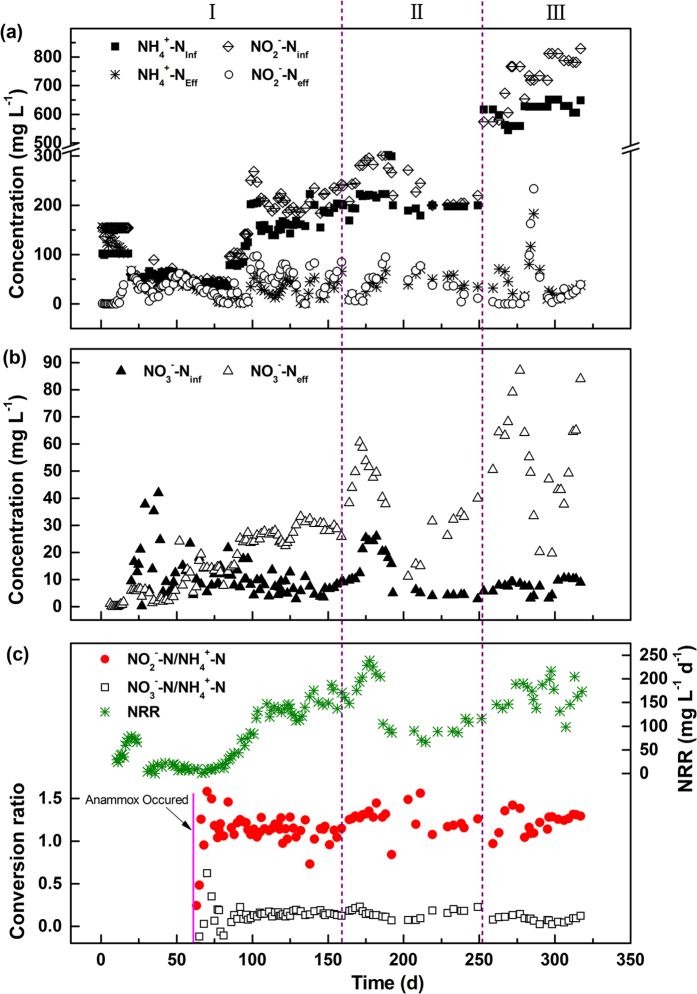
Nitrogen removal performance in the anammox reactor. (**a**) influent and effluent ammonium and nitrite concentrations; (**b**) influent and effluent nitrate concentrations; (**c**) NRR, conversion ratios of nitrite to ammonium, and ratio of nitrate production to ammonium conversion.

The anammox reactor went through three periods: startup (days 1–159), acclimatization to raw leachate (days 160–249) and operation with raw leachate (days 250–317). The concentrations of ammonium, nitrite and nitrate in the reactor, NRR, as well as the conversion ratio of nitrite to ammonium and the ratio of nitrate production to ammonium conversion are presented in Fig. 3. In the startup period (Period I), the anammox reactor was continuously fed with synthetic wastewater containing ammonium and nitrite in the required amounts. Based on the effluent ammonium concentration, the anammox process was divided into the phases of sluggish (days 1–20), expressive (days 21–60), enhanced (days 61–103) and steady (days 104–159). In the sluggish phase, the ammonium concentration in the effluent was clearly higher than that in the influent. This phenomenon might result from the bacteria cell lysis and the breakdown of organic nitrogen in the seed sludge to ammonium^30^. Moreover, nearly all of the nitrite in the influent was removed indicating that heterotrophic denitrification was the dominant process. In the expressive phase, the anammox activity appeared, as the ammonium and nitrite concentrations in the effluent decreased simultaneously. In the enhanced phase, the ammonium and nitrite concentration exhibited a rapid decline in the effluent. NRR gradually increased from 9 mg N L^−1^ d^−1^ to 147 mg N L^−1^ d^−1^. In the steady phase, the anammox activity exhibited a good stability, and a NRR of 143 ± 20 mg N L^−1^ d^−1^ was obtained, marking a successful start-up of anammox process. The startup phase lasted for 103 days which was close to that reported in UASB^31^ and SBR^30^ but longer than that in MBR^32^. The NRR obtained in the study was higher than those reported in UASB and SBR (40–90 mg N L^−1^ d^−1^)^31^,^33^, but lower than that in MBR (345 mg N L^−1^ d^−1^)^32^. The discrepancy could be probably attributed to the different sources of seed sludge. The conversion ratio of nitrite to ammonium was 1.12 ± 0.12, which was slightly lower than the previously reported values of 1.32^7^. This could be partly because of the activities of other bacteria, such as AOB living on the leakage of oxygen into the MBR^32^. Also, the ratio of nitrate production to ammonium conversion was 0.15 ± 0.02, whereas the theoretical ratio was 0.26. A possible explanation for the low nitrate concentration could be that a small part of denitrifying bacteria surviving in the reactor might reduce nitrate produced by AMX^31^.

In the acclimatization period (Period II), NRR gradually increased to the highest value of 238 mg N L^−1^ d^−1^ on day 173 when the proportion of PN effluent in the feed was increased to 20%. Meanwhile, the effluent ammonium and nitrite concentrations decreased to lower than 10 mg N L^−1^. When the proportion was further increased to 50%, the NRR decreased to less than 100 mg N L^−1^ d^−1^. However, the reactor was able to recover its performance after a long inoculation period. The results indicated that a lower proportion of landfill leachate could promote the anammox activity, whereas a higher proportion may inhibit the anammox activity. However, the AMX could adapt to the higher proportion after a long period of inoculation.

In Period III, the reactor was operated with real PN effluent. The ammonium, nitrite and nitrate concentrations in the PN effluent were kept at 611 ± 34, 727 ± 82 and 8 ± 2 mg N L^−1^, respectively. During this period, the effluent ammonium, nitrite and nitrate concentrations were 30 ± 20, 15 ± 15 and 56 ± 20 mg N L^−1^, respectively, except on days 312–320. On days 312–320, in order to further improve the nitrogen removal, sodium acetate at an equivalent concentration of approximately 300 mg L^−1^ COD was supplemented to denitrify the produced nitrate. However, the effluent ammonium and nitrite concentration increased greatly. One possible explanation for the decreased anammox activity was that, when organic carbon coexisted with ammonium and nitrite, the heterotrophic denitrifiers would grow at a higher rate, thus anammox bacteria could not compete with heterotrophic denitrifiers for both space and electron acceptor (nitrite)^34^. Another possible explanation could be that easily biodegradable COD promoted the sulfate reduction to sulfide, which in turn inhibited AMX activity. Jin, *et al*.^35^ reported that the performance of the anammox granular sludge was halved at a sulfide level of 32 mg S L^−1^. In this study, the inhibition threshold value could be much lower owing to a lower amount of anammox biomass in the reactor. As organic matter was removed from the influent, the anammox recovered quickly with an NRR of 166 ± 30 mg N L^−1^ d^−1^ probably owing to the strong biomass retention capacity of MBR, although it was somewhat lower than that obtained prior to COD addition. The conversion ratio of nitrite to ammonium was 1.22 ± 0.12, and the ratio of nitrate production to ammonium conversion was 0.08 ± 0.03, indicating that some organic matter in the PN effluent promoted the survival of a small portion of denitrifying bacteria in the reactor.

The obtained results indicated that the operation of the anammox process in the MBR was suitable for landfill leachate treatment. After operating for nearly one year, the maximum NRR of the anammox process was close to the values obtained using floc-based reactors and lower than those obtained from the granule-based reactors described in the literature. Wang, *et al*.^32^ reported that a maximum NRR of approximately 320 mg N L^−1^ d^−1^ could be achieved for synthetic wastewater treatment in the MBR. Ruscalleda, *et al*.^36^ reported that an average NRR of 240 mg N L^−1^ d^−1^ with nitrogen removal efficiencies of up to 89% was obtained for landfill leachate treatment in an SBR. Lan, *et al*.^37^ reported that an NRR of 62 mg N L^−1^ d^−1^ was obtained in a floc-based SNAD in an SBR. However, an NRR of 382 mg N L^−1^ d^−1^ could be achieved in a granule-based SNAD in a full-scale bioreactor treating mature landfill leachate^1^. Granule-based reactors typically lead to high nitrogen removal. Some studies were able to reach a NRR of over 7000 mg N L^−1^ d^−1^ for municipal wastewater treatment in a UASB with granule biomass after operating for several years^38^. In this study, a maximum NRR of 216 mg N L^−1^ d^−1^ for landfill leachate treatment was achieved through the anammox process. Apart from heterotrophic denitrifiers competition and the sulfide inhibition, the presence of heavy metals in site-specific landfill leachate might also inhibit the rapid increase of NRR. It was reported that AMX were highly sensitive to heavy metal toxicity with 50% inhibiting soluble concentrations of 3.9–7.6 mg L^−1^ and 1.9–14.5 mg L^−1^ by zinc and copper, respectively^39^,^40^. The nitrogen removal performance of anammox process could be further enhanced by the enrichment of more anammox biomass and by identifying the inhibitory factors in the landfill leachate.

### Microorganisms in the anammox reactor

The qPCR analysis of seed sludge and sludge samples collected from the anammox reactor on days 61, 86 and 277 (Table 1) showed that the total bacterial 16S rRNA gene number decreased sharply from 2.97 ± 0.36 × 10^12^ in the seed sludge to 1.28 ± 0.16 ×10^12^ copies L^−1^ on day 66, after which it stabilized. In comparison, the AMX *hzo* gene and 16S rRNA gene copy numbers increased significantly after inoculation. The *hzo* gene exhibited the lowest copy number in seed sludge (9.28 ± 2.27 × 10^8^ copies L^−1^). On day 23, the copy number of the *hzo* gene was only three-fold higher than that in the seed sludge. However, by days 86 and 277, the copy numbers of *hzo* genes had increased to 5.39 ± 0.40 × 10^10^ and 1.54 ± 0.015 × 10^11^ copies L^−1,^ representing approximately 59 times and 166-fold increases, respectively, compared to the copy numbers in the seed sludge. Furthermore, the ratio of the copy number of the *hzo* gene to that of the total bacterial 16S rRNA genes increased from 0.03% in the seed sludge to 11.46% in the enrichment sludge on day 277. A similar trend could be observed from the qPCR analysis of AMX 16S rRNA gene.

On days 0, 61, 86 and 277, the NRR was calculated to be approximately 0, 4, 72 and 190 mg N L^−1^ d^−1^, respectively (Fig. 3c). Based on the linear regression analysis of the NRR and gene copy number, the NRR was significantly related to both the *hzo* gene and AMX 16S rRNA gene (R^2^ = 0.97). The cell-specific anammox activity was approximately 68–95 fmol N cell^−1^ d^−1^, assuming that one AMX cell contains one copy of the *hzo* gene. This activity is approximately twice as high as the anammox activity (33–37 fmol N cell^−1^ d^−1^) reported in 18-month anammox enrichment cultures of a flooded paddy soil^41^ and considerably higher than the activities found in natural environments (2.9–21 fmol N cell^−1^ d^−1^)^42^.

### Implication of the landfill bioreactor-PN-anammox process

**Figure 4:**
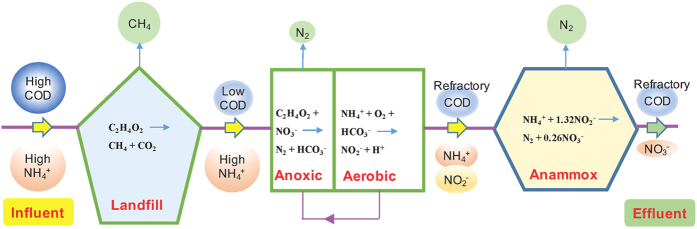
The pathways for methane recovery and nitrogen removal in the combined landfill bioreactor, partial-nitritation and anammox process.

In this study, the integrated landfill bioreactor-PN-anammox process has been established and proven to be suitable for high-strength landfill leachate treatment (Fig. 4). Traditionally, landfill bioreactor has been considered to decrease leachate organic strength and increase waste degradation rates via *in-situ* leachate recirculation. However, ammonium nitrogen remains a big issue. In the developed technology, simultaneous *in-situ* organic mater degradation and *ex-situ* efficient nitrogen removal could be achieved. Additionally, the combined process demonstrated the superiority over conventional landfill leachate treatment technologies in terms of energy savings. Anaerobic landfill bioreactor could convert organic matter to methane generally characterized by low operational costs (energy), while PN-anammox process could save up to 2/3 energy compared to conventional nitrification/denitrification process. Moreover, owing to a strong biomass retention capacity with little concern about sludge flotation or wash-out, the anammox MBR process showed an effective startup, as well as a quick recover from an inhibitory condition. Nitrogen removal rate of the anammox MBR in the present study was a little lower than that (about 250‒680 mg N L^−1^ d^−1^) in conventional partial nitrification/denitrification process^25^,^43^, which could be further enhanced by accumulation of more anammox cell concentrations. Thus, it suggests that the application of the combined process to landfill leachate treatment was very promising. When applying the combined process, several questions are raised and attentions need to be paid. First of all, to obtain high-efficient nitrogen removal via anammox, the majority of biodegradable COD should be removed prior to anammox reactor to avoid the competition between heterotrophic denitrifiers and AMX. Secondly, the effluent from the integrated process still contains high concentrations of refractory organic compounds and TN. To meet more stringent nitrogen discharge standard for landfill leachate imposed globally by the regulating agencies, a combined advanced oxidation process (such as Fenton, ozonation, UV irradiation, ozone combined with UV or H_2_O_2_^44^) and biological methods are needed for post-treatment. Thirdly, anaerobic MBR should be further optimized for anammox application to minimize the membrane fouling and maintain long-term continuous operation. In addition, the composition of landfill leachate exhibits noticeably temporal and site-specific variation, so it is necessary to take into account the heterogeneous composition of landfill leachate with different landfill ages and sites.

## Methods

### Reactor set-up

**Figure 5:**
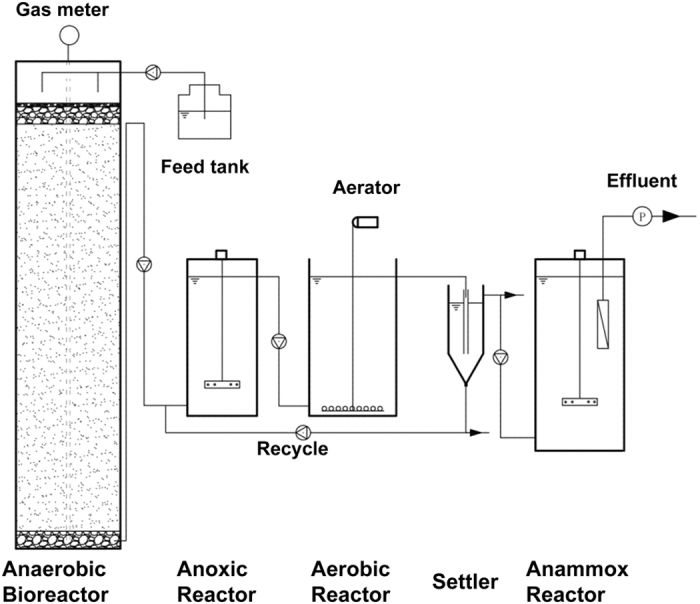
Schematic diagram of the combined landfill bioreactor, partial-nitritation and anammox process.

The experimental system presented in Fig. 5 was comprised of a landfill bioreactor, anoxic reactor, aerobic reactor and anammox reactor. The landfill bioreactor (29 cm diameter, 115 cm height) had an effective volume of 76 L and a working volume of 11 L. A 20 cm-thick layer of 2 cm-diameter gravel was placed at the bottom of the reactor. The anoxic reactor (18 cm diameter; 45 cm height) had an effective volume of 7 L, and complete mixing was achieved by a mechanical stirrer at a speed of 100 rpm. The aerobic reactor (25 cm length; 25 cm width; 45 cm height) had an effective volume of 22 L, and aeration was provided via fine bubble air diffusers. The settler had an effective volume of 3 L. The anammox reactor (30 cm length; 25 cm width; 55 cm height) had an effective volume of 32 L and was sealed tightly to maintain an anaerobic condition. A submerged flat-sheet membrane module was placed within the reactor to ensure the complete retention of the suspended bacteria. The membrane (pore size: 0.1 μm) was made of polyvinylidene fluoride with a total area of 0.1 m^2^. The sludge was fully mixed by a mechanical stirrer at a speed of 50 rpm.

### Operational conditions

The landfill bioreactor was loaded with 7-year-old landfill waste, which was excavated from the Tianziling Landfill in China. Approximately 65.3 kg of landfill waste was loaded into the reactor at a solid packing density of 860 kg m^−3^. The characteristics of refuse were as follows: pH 8.21 ± 0.15, water content 32.0 ± 4.2%, TN 3.1 g kg^−1^, TP 1.8 g kg^−1^, volatile solids 268.5 ± 0.6 g kg^−1^. The anoxic reactor, aerobic reactor and anammox reactor were inoculated with activated sludge which was collected from the landfill leachate treatment plant in Tianziling Landfill, China. The inoculated sludge showed a good performance performing simultaneous nitrification and denitrification. After inoculation, the concentrations of total suspended solids (TSS) in the anoxic reactor, aerobic reactor and anammox reactor were 10000, 8000 and 26000 mg L^−1^, respectively, and the VSS/TSS (VSS: volatile suspended solids) was approximately 0.5. After inoculation, each reactor was operated for approximately 100 days to enrich with different functional groups (methanogens, denitrifiers and nitrifiers). AMX were individually enriched in the anammox reactor because AMX grow slowly and the startup time is long^45^. The startup of anammox reactor was successfully achieved in 159 days through the sluggish, expressive, enhanced and steady phases. It was continuously fed with synthetic wastewater containing ammonium and nitrite in the required amounts. Additionally, the wastewater contained a growth medium and trace nutrient solution as described by Chen, *et al*.^31^. The nitrogen loading rate (NLR) was enhanced by increasing the concentrations of ammonium and nitrite in the feed.

After the enrichment period, the experimental system was operated in a continuous feeding mode. In the landfill bioreactor, efficient COD removal and methane recovery were accomplished by an anaerobic methanogenic process prior to the biological nitrogen removal process. In the subsequent anoxic/aerobic reactor, pre-denitrification occurred in the anoxic reactor where a fraction of organic matter was degraded. PN and the effluent NO_2_^−^/NH_4_^+^ molar ratio of approximately 1 were chosen in the aerobic reactor to obtain a suitable effluent to anammox, whereas nitrite was recycled to the anoxic reactor by partial recirculation. The recycling ratio, expressed as the ratio between the flow of recycled liquor and the inlet flow, was equal to 1.

Experiments measuring the effects of alkalinity on the effluent NO_2_^−^/NH_4_^+^ molar ratio in the aerobic reactor were carried out in three phases: Phase i, from day 1 to day 23, the aerobic reactor was fed with anaerobic/anoxic treated leachate and PN was obtained; Phase ii, from day 24 to day 198, the long-term operation of oxidizing approximately half of the ammonium to nitrite was conducted by adjusting the alkalinity concentrations; and Phase iii, from day 200 to day 250, the aerobic reactor was again fed with pre-treated leachate.

In the anammox reactor, three experimental periods were used to investigate the feasibility of anammox activity treating landfill leachate. During Period I, the reactor was initially operated with synthetic wastewater. During Period II, the reactor was fed with a mixture of synthetic wastewater and PN effluent. The proportion was progressively increased (from 10% to 50% v v^−1^), until reaching 100% of PN effluent. During Period III, the reactor was operated for 67 days with real PN effluent without diluting the composition.

The temperature of the mixed liquor in the landfill bioreactor, anoxic reactor and aerobic reactor was maintained at 28 ± 1 °C. The anammox reactor was operated at 31 ± 1 °C using a water jacket.

### Landfill leachate characteristics

Landfill leachate was obtained from the Tianziling Landfill in Hangzhou, China. The characteristics of the raw leachate are presented in Table 2.

### Chemical analysis and calculations

COD, BOD_5_, ammonium, nitrite, nitrate, TP and TN were measured according to the standard methods^46^. The pH and DO concentration were measured with a pH meter (SG2, Mettler-Toledo, Greifensee, Switzerland) and an oxygen probe (YSI 550A, YSI, OH, USA), respectively. Alkalinity was measured by titrating against a standard HCl solution. TSS and VSS were measured by the weighing method after being dried at 105 °C and burnt to ash at 550 °C. The volume of the produced biogas was determined by a wet-test gas flow meter (LMF-1, Changchun, China), whereas the biogas composition was analyzed via a gas chromatograph (GC9800, Shanghai, China) coupled with a thermal conductivity detector^47^. NRR was calculated based on TN removed (ammonium, nitrite and nitrate) per unit of reactor volume and time. Free ammonia concentration was calculated according to the following equation:


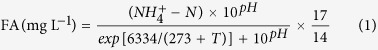


### Quantitative polymerase chain reaction (qPCR) analysis

The abundance of AMX was quantified in triplicate via SYBR green chemistry qPCR, specifically targeting the AMX 16S rRNA gene and hydrazine oxidoreductase (*hzo*) gene. A primer set (Amx 368F/820R) targeting the 16S rRNA gene was used to quantify AMX^48^. The qPCR primers (hzocl1F1/hzocl1R2) were designed to target the *hzo* gene of AMX^49^. Total bacterial abundance was also quantified using universal 16S rRNA targeted primers (338F/518R)^50^. qPCR was performed on a Bio-Rad CFX96 system (Bio-Rad, Inc., Hercules, CA, USA), and the operation procedure was based on the method described by Chen, *et al*.^31^. The PCR program for *hzo* gene was as follows: denaturation for 3 min at 95 °C, followed by 39 cycles of 10 s at 95 °C, annealing for 30 s at 50.6 °C and elongation for 30 s at 72 °C. All real-time PCR assays were performed using three replicates per sample, and all PCR runs included control reactions without the template. The gene copy numbers were calculated by comparing threshold cycles obtained in each PCR run with those of known standard DNA concentrations. Standard curves were obtained using serial dilutions of linearized plasmids containing cloned *hzo*, and AMX 16S rRNA genes.

## Additional Information

**How to cite this article**: Sun, F. *et al*. Integrating landfill bioreactors, partial nitritation and anammox process for methane recovery and nitrogen removal from leachate. *Sci. Rep.* **6**, 27744; doi: 10.1038/srep27744 (2016).

## Supplementary Material

Supplementary Information

## Figures and Tables

**Table 1:** Copy numbers of the *hzo* and 16S rRNA genes of AMX and the 16S rRNA gene of total bacteria in sludge samples of the anammox reactor during the entire operation.

Sludge sample	Gene copy number per liter of mixed liquor (copies L^−1^)			Copy number ratio (%)	
	Bacterial 16S rRNA gene	*hzo*	AMX (16S rRNA)	*hzo*to 16S rRNA gene	AMX to 16S rRNA gene
Seed	2.97 ± 0.36 × 10^12^	9.28 ± 2.27 × 10^8^	8.88 ± 0.16 × 10^7^	0.03	0.003
Day 61	1.28 ± 0.16 × 10^12^	3.16 ± 0.88 × 10^9^	1.63 ± 0.16 × 10^8^	0.25	0.01
Day 86	1.28 ± 0.04 × 10^12^	5.39 ± 0.40 × 10^10^	9.77 ± 3.17 × 10^10^	4.22	7.64
Day 277	1.34 ± 0.26 × 10^12^	1.54 ± 0.15 × 10^11^	1.82 ± 0.05 × 10^11^	11.46	13.61

**Table 2:** Characteristics of the landfill leachate during the experiments.

Item	Range
pH	6.87–8.53
Conductivity (mS cm^−1^)	13.47–16.35
Alkalinity as CaCO_3_, mg L^−1^	11100–16000
COD (mg L^−1^)	1532–12633
BOD_5_ (mg L^−1^)	713–648
NH_4_^+^-N (mg L^−1^)	953–3340
PO_4_^3−^-P (mg L^−1^)	9.9–26.2
