# Supplementary material for: Integrating landfill bioreactors, partial nitritation and anammox process for methane recovery and nitrogen removal from leachate
Source: Sci Rep. 2016 Jun 9;6:27744. doi: 10.1038/srep27744 (PMC4899737; doi:10.1038/srep27744)
Supplement: Supplementary Information [file srep27744-s1.pdf]

Integrating landfill bioreactors, partial nitritation and anammox process for methane recovery and nitrogen removal from leachate

Faqian Sun [a], Xiaomei Su [a], Tingting Kang [a], Songwei Wu [b], Mengdong Yuan [a], Jing Zhu [a], Xiayun Zhang [c], Fang Xu [c], Weixiang Wu [a].

[a] Institute of Environmental Science and Technology, Zhejiang University, Hangzhou 310058, China

[b] Quzhou Environmental Sanitation Department, Quzhou 324000, China

[c] Zhejiang Institute of Microbiology, Hangzhou 310012, China

*Corresponding author: Weixiang Wu

Address: Yuhangtang Road 866#, Hangzhou 310058, China

E-mail: weixiang@zju.edu.cn

Tel: +86-571-88982020

Fax: +86-571-88902020

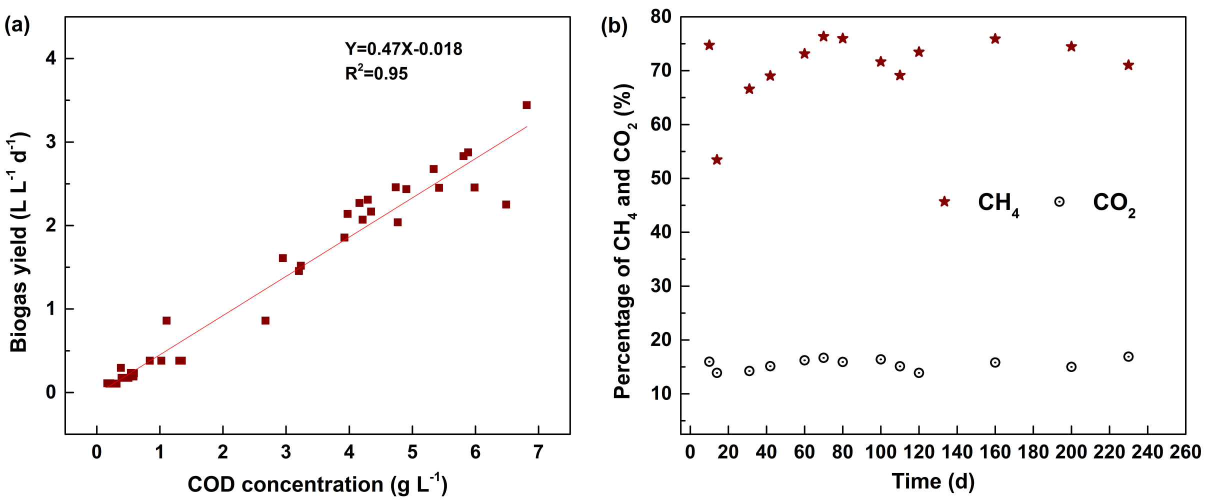

Supplementary Figure S1 (a) The relationship between COD removal and biogas production. (b) Profiles of CH_4 emission

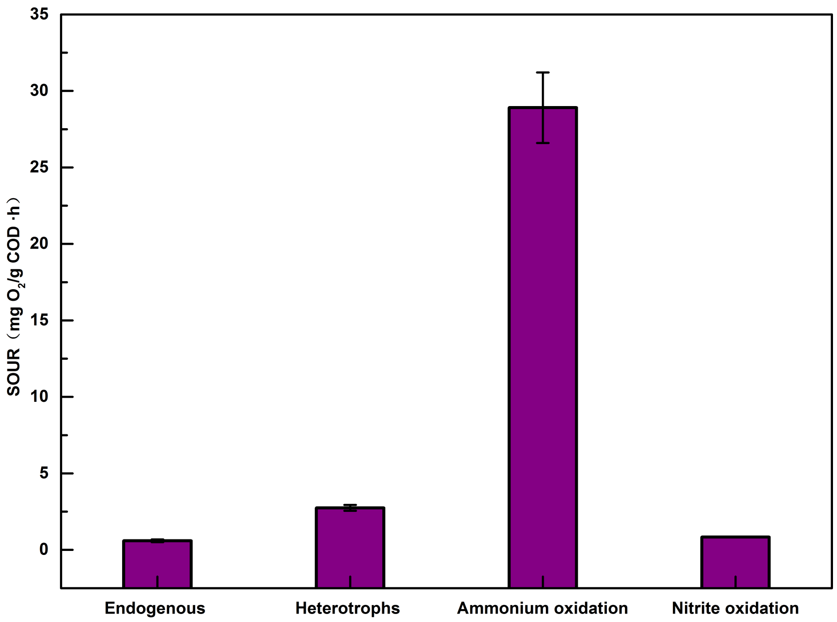

Supplementary Figure S2 Specific oxygen uptake rate (SOUR) of the different types of respiration on day 100. SOUR was measured in duplicate using batch extant respirometric assays described by Surmacz-Gorska et al.[1]. Biomass concentration was measured using HACH COD kits.

1 Surmacz-Gorska, J., Gernaey, K., Demuynck, C., Vanrolleghem, P. & Verstraete, W. Nitrification monitoring in activated sludge by oxygen uptake rate (OUR) measurements. *Water Res.* **30**, 1228-1236 (1996).
